# Supplementary material for: The Construction and Exploration of a Comprehensive MicroRNA Centered Regulatory Network in Foxtail Millet (Setaria italica L.)
Source: Front Plant Sci. 2022 May 6;13:848474. doi: 10.3389/fpls.2022.848474 (PMC9121102; doi:10.3389/fpls.2022.848474)
Supplement: Supplementary file 2 [file Data_Sheet_2.zip › Sit-miR396a_Seita.1G271800.1_648_TPlot.pdf]

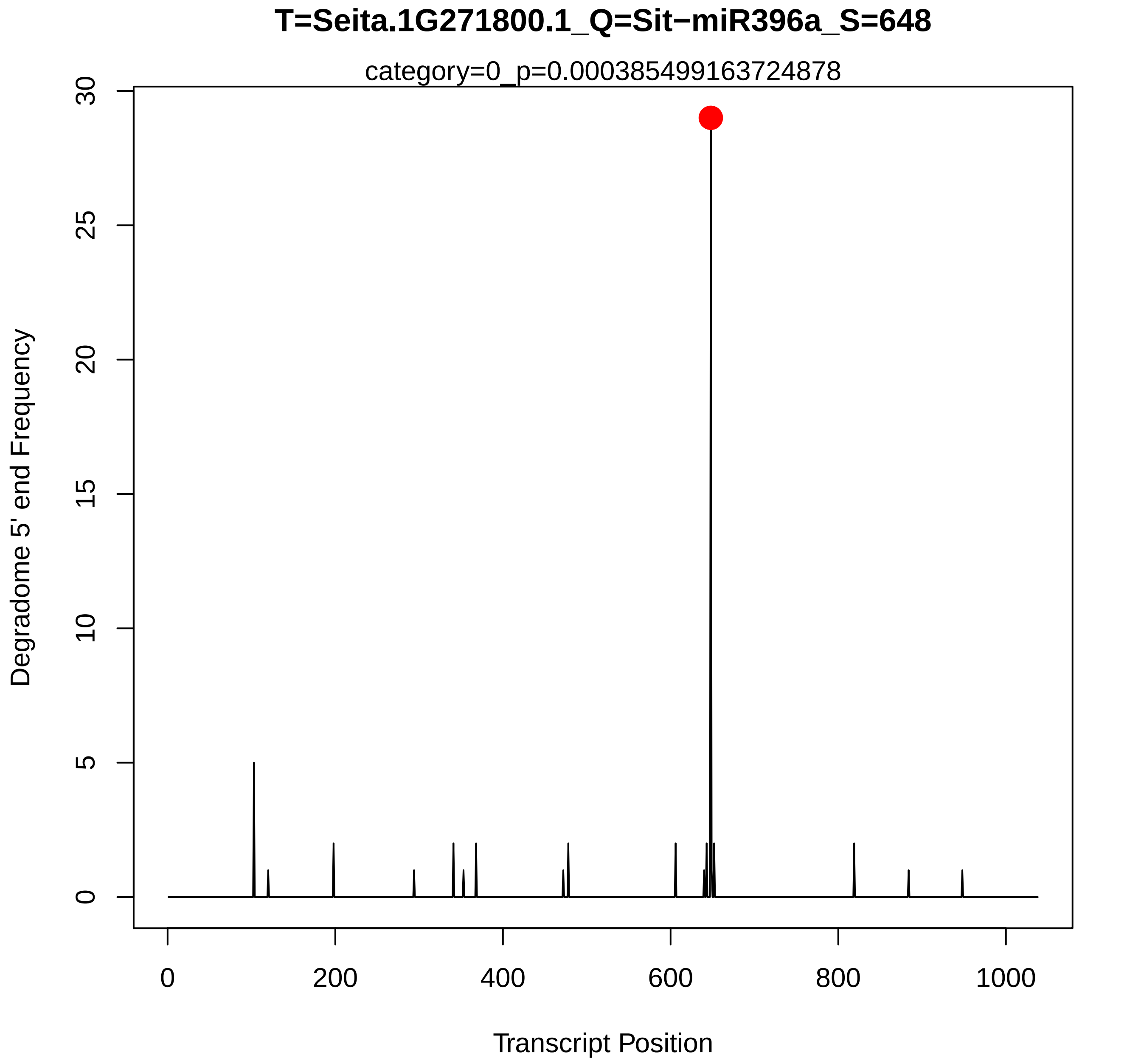
T=Seita.1G271800.1_Q=Sit−miR396a_S=648
category=0_p=0.000385499163724878
Degradome 5' end Frequency
0
5
10
15
20
25
30
Transcript Position
0
200
400
600
800
1000
